# Inflammaging in Endemic Areas for Infectious Diseases

**DOI:** 10.3389/fimmu.2020.579972

**Published:** 2020-11-12

**Authors:** Marina Andrade Batista, Fernanda Calvo-Fortes, Gabriela Silveira-Nunes, Giovanna Caliman Camatta, Elaine Speziali, Silvia Turroni, Andrea Teixeira-Carvalho, Olindo A. Martins-Filho, Nicola Neretti, Tatiani Uceli Maioli, Rodrigo Ribeiro Santos, Patrizia Brigidi, Claudio Franceschi, Ana Maria Caetano Faria

**Affiliations:** ^1^ Programa de Pós Graduação em Nutrição e Saúde, Escola de Enfermagem, Universidade Federal de Minas Gerais, Belo Horizonte, Brazil; ^2^ Departamento de Medicina, Universidade Federal de Juiz de Fora, Governador Valadares, Brazil; ^3^ Instituto Rene Rachou, Fundação Oswaldo Cruz, Belo Horizonte, Brazil; ^4^ Unit of Microbial Ecology of Health, Department of Pharmacy and Biotechnology, University of Bologna, Bologna, Italy; ^5^ Departament of Molecular Biology, Cell Biology and Biochemistry, Brown University, Providence, RI, United States; ^6^ Departamento de Clínica Médica, Faculdade de Medicina, Universidade Federal de Minas Gerais, Belo Horizonte, Brazil; ^7^ Center for Biophysics, Bioinformatics, Biocomplexity, University of Bologna, Bologna, Italy; ^8^ Laboratory of Systems Biology of Healthy Aging, Department of Applied Mathematics, Lobachevsky University, Nizhny Novgorod, Russia; ^9^ Departamento de Bioquímica e Imunologia, Instituto de Ciências Biológicas, Universidade Federal de Minas Gerais, Belo Horizonte, Brazil

**Keywords:** aging, inflammation, chronic infection, genetics, microbiota, dietary components, inflammaging

## Abstract

Immunosenescence is marked by a systemic process named inflammaging along with a series of defects in the immunological activity that results in poor responses to infectious agents and to vaccination. Inflammaging, a state of low-grade chronic inflammation, usually leads to chronic inflammatory diseases and frailty in the elderly. However, some elderly escape from frailty and reach advanced age free of the consequences of inflammaging. This process has been called immunological remodeling, and it is the hallmark of healthy aging as described in the studies of centenarians in Italy. The biological markers of healthy aging are still a matter of debate, and the studies on the topic have focused on inflammatory *versus* remodeling processes and molecules. The sub-clinical inflammatory status associated with aging might be a deleterious event for populations living in countries where chronic infectious diseases are not prevalent. Nevertheless, in other parts of the world where they are, two possibilities may occur. Inflammatory responses may have a protective effect against these infectious agents. At the same time, the long-term consequences of protective immune responses during chronic infections may result in accelerated immunosenescence in these individuals. Therefore, the biological markers of healthy aging can vary according to environmental, cultural, and geographical settings that reflect worldwide, and in a non-biased, non-westernized perspective, the changes that we experience regarding our contacts with microorganisms and the outcomes of such contacts.

## Introduction

Inflammaging, which has been described as a state of low-grade chronic inflammation associated with dysfunctional immunity, is the hallmark of immunosenescence. The consequences of both processes may lead to increased susceptibility to infection and poor responses to vaccination as well as to chronic inflammatory and degenerative diseases in the elderly. However, a critical observation coming from the studies of centenarians in Italy was that some aged individuals reach this advanced age without chronic diseases or frailty. These studies showed that unlike frail individuals, healthy European centenarians have immune-modulatory mechanisms that compensate for the inflammaging and prevent the development of chronic inflammatory diseases (1). On the other hand, many individuals living in endemic areas of infectious diseases in developing countries manage to stay clear of infection throughout life due to remodeling mechanisms of innate immunity that could be classified as inflammatory. Nevertheless, chronic exposure to infectious agents and the protective mechanisms needed to cope with it can also function as aging acceleration stimuli.

The concept of healthy aging was proposed in Europe to describe individuals who reach advanced age free of inflammatory consequences of immunosenescence (1, 2). However, this concept might be incomplete because it does not take into account environmental and geographical differences that would interfere with the effects of inflammaging. Understanding the immunological and biological consequences of living in areas where contact with infectious agents is continuous and of high intensity may provide valuable elements to broaden the concept of healthy aging.

## Senieur Protocol, Criticisms, and Criteria to Recognize Aging as Healthy

The description of inflammaging as a major event in immunosenescence has fostered a growing interest among researchers and physicians in its effects on age-related diseases and on healthy aging, as its counterpart (3–6). Not only the number of elderlies is increasing around the world, but today they expect to live much longer (7). In the case of Brazil, recent data shows that the country has experienced an unprecedent demographic process of aging of its population when compared to more developed countries although it is not clear which is the real size of elderly population (specially centenarians) due to incorrect recording of age over the years and the quality of data (8). In this scenario, a first challenge we still have to face is to define what healthy aging is. A misinterpretation of any age-associated condition as age-determined can alter study results and its usefulness.

The first results on the immunological alterations brought about by aging were conflicting due to bias in patient selection, which was one of the reasons for the creation of the SENIEUR protocol (9). This protocol was proposed in 1984 by Ligthart and coworkers to better distinguish immunosenescence from age-associated diseases, and it consists of a set of criteria in which clinical information as well as laboratory data are evaluated. Any overt disorder that might influence the immune system should be excluded, including Crohn’s disease, collagen-vascular diseases, tumors, and infections. This generated a uniformity in patient selection for immunological studies and was a huge achievement. In the same period, most geriatric clinical studies used different criteria for normal aging dividing it into usual aging (when extrinsic factors accentuate the effects of aging) and successful aging (when extrinsic factors play a neutral role). Successful aging was defined as absence or low risk of disease, high functionality, and high engagement with life (10, 11).

According to Castle and coworkers (12), 16 years after its creation, SENIEUR protocol still proved to be methodologically viable because it was able to reveal immunological differences between “healthy elderly” (who fit the SENIEUR protocol) and “almost healthy elderly” (who nearly fit). However, stringent criteria are the SENIEUR protocol strength and weakness. Some studies showed that this protocol may exclude a significant proportion of the elderly living independently at home and leading an active life (12, 13).

A different approach to evaluate the immune system emerged from data obtained in immune longitudinal studies enrolling oldest-old subjects such as OCTO and NONA studies (14). They described octogenarians and nonagenarians who had mild chronic diseases in spite of their longevity and reported that morbidity did not significantly impact on the T-cell immune risk phenotype (15). In addition, an American centenarian cohort study revealed that 24% male and 43% women fit in the survivor morbidity profile (*i.e.*, centenarian patients who had a diagnosis of an age-associated illness prior to the age of 80) (16). Probably, the huge majority of them would not be considered healthy elderly by SENIEUR protocol. Since some experts consider centenarians as the best model to study human longevity (17), this data posed doubts on the fitness of the SENIEUR protocol for understanding longevity.

The idea of disease-free elderly subjects and the exclusion of any condition that might influence the immune system, as imposed by SENIEUR protocol, narrow the attention to a very exclusive group of healthy seniors (18) who are not representative of the elderly population. Apart from that, this protocol should be continuously updated as laboratory data and diagnosis mature and also does our knowledge about age-associated diseases. Now it is widely accepted that degenerative diseases usually start many years or even decades before they become clinically apparent, and immunological alterations are present much earlier than clinical symptoms (19–21). Pre-clinical diagnosis for some conditions (such as Alzheimer disease) are also already part of clinical and research practices. Therefore, as time passed and technological advances allowed early diagnosis of these pathological conditions, elimination of the ‘multimorbidity noise’ when examining immunosenescence became increasingly difficult.

Although our knowledge about diseases has increased during the last decades, no augment in life span and health span was observed by singly studying them. Aging still is the main risk factor for chronic diseases. In order to have a better “bench-to-bedside” approach, it is important to be more inclusive, and aging should be studied in association with chronic conditions. Geroscience was created in this scenario as the intersection between the biology of age-related chronic diseases and the basic biology (22). As science developed so did the concept of what is normal aging. The presence of a disease says little about the impact it may have on an older person’s life. Health cannot be viewed also as simply the absence of diseases. The World Health Organization defines Healthy Ageing “as the process of developing and maintaining the functional ability that enables wellbeing in older age” (7). From an immunological point of view, the concept of healthy aging has been proposed to describe the “individuals who reach advanced age free of inflammatory consequences of senescence” (23, 24). Unfortunately, there is no good biomarker for senescence which incorporates other elements strongly correlated with aging—autophagy, mitochondrial function, cellular senescence, and DNA methylation (25). In addition, all these mechanisms are linked in a complex and dynamic network to maintain homeostasis (25). Thus, a broader approach when studying the pace of senescence will certainly be more fruitful to understand the mechanisms that trigger or remodel the alterations brought about by aging itself.

Aligned with the concept of heathy aging and geroscience, our group use the clinical-functional categorization (26) classifying elderly into three categories (*i.e.*, robust, at risk of frailty, and frail). These three categories encompass 10 sub-categories. According to this categorization, individuals who are fully independent (*i.e.*, able to perform advanced activities of daily life), autonomous, do not have sarcopenia, mild cognitive impairment, frailty syndrome (27) or complex multi-morbidity are considered robust.

Finally, it is important to stress that, depending on the research question addressed by a particular aging study, the choice of a healthy elderly control group may vary but the inclusion and exclusion criteria for a patient to be classified as reference group should be clearly stated.

## Aging, Inflammaging, and Remodeling 

Although aging is a physiological process characterized by several changes in the organism as a whole, this review is focused on immunosenescence since the alterations in the function of the immune system impact on other organs and tissues (28). Immunosenescence can be described as a complex and multifactorial process influenced by genetic and microenvironment that results in gradual decrease of immunological activities including effector responses and their regulation (4, 28). Therefore, aging is associated with increased vulnerability to infectious and chronic diseases, and impairment of immune responses to vaccination (29–33). Investigations on the age-related changes occurring in the immune system of different populations (Sweden, Holland and Belgium) suggested that the immune parameters associated with mortality in the elderly are context-dependent (34). Thus, a major role in immunosenescence is played by the environmental conditions. We have conceptualized this main characteristic of immunosenescence by proposing the new concept of “immunobiography”, defined as the combination of type, dose, intensity, and temporal sequence of antigenic stimuli that each individual is exposed throughout life (35). Owing to its memory and plasticity, the immune system is capable of adapting and recording all these immunological experiences. The immunological history of each individual is responsible for the capability in single persons to mount strong, weak, or no response to specific antigens, thus determining the large heterogeneity of immunological responses observed in the elderly (35).

Several immunological alterations associated with aging are well described in the literature (**Table 1**), and they heavily impact the T cell compartment (29, 36). Thymic involution is a hallmark of immunosenescence responsible for the early decline in the output of naïve T cells to the peripheral blood and, consequently, for the shrinking of the T cell repertoire (29, 30). Indeed, a universally observed aging-associated immunological alteration is the decrease of naive T cells (particularly CD8+ T cells) in the peripheral blood (37). Concomitantly, chronic stimulation of the immune system results in the increase of peripheral CD4+ and CD8+ memory T cells (38). Furthermore, the inverted CD4/CD8 ratio that was identified in aged individuals has been associated with increased frequencies of terminal memory T cells and of senescent exhausted lymphocytes (expressing PD-1, KLRG-1, CD57, TIM-3) with low proliferative capacity, defects in signaling pathways, and loss of molecules necessary for co-stimulation such as CD28 and CD27 (31, 32, 36). The aging-related shift in the bone marrow maturation of hematopoietic cells towards myelocytic differentiation (39) results in a decrease in naïve B cell production and an increasing oligoclonal B cell repertoire over a span of decades (64). The lower production of naive B and T cells and the alterations in their repertoire diversity and their interactions result ultimately in a poor ability to trigger effective responses against novel antigens (33, 40).

**Table 1:** Summary of some changes during immunosenescence.

Compartment	Overall changes	References
**Adaptive immunity**		
Decrease in Naïve cell number Decrease in IL-2 production Decrease in lymphoid number Decrease in CD27 expression Decline in antibody diversity (B cells) Increase in memory cell number Increase in regulatory T cell number Increase in CD8+CD28− T cells	Shrinkage of T cell receptor repertoire; Loss of immunological space; Less responsive to immune stimulation/infection and vaccination; Less efficient responses to stress; Decrease ability to cope with environmental challenges, such as reactivation of chronic and new infections; Accumulation of senescent cells in tissue and organs; Reduced proliferative capacity of T cells; Autoimmunity; Immune dysfunctions	(1–4, 24, 29, 30, 32, 33, 36–52)
**Innate immunity**		
Increase in cytokine production Increase in myeloid number Increase in NK cells Increase in the activity of certain signaling pathways, while other pathways are impaired Decrease or no change in phagocytosis Decrease in chemotaxis	Chronic progressive increase in the pro-inflammatory status resulting in inflammaging; Tissue damage and organ dysfunctions; Delayed wound healing; Loss of homeostasis; Modification of interaction with T cells; Predisposition to diseases and risk of frailty	(5, 6, 28, 31, 35, 39, 44, 51, 53–63)

IL, interleukin; Nk cells, Natural killer cells.

At the same time, it is known that senescent cells, in spite of the progressive loss of their activity and proliferative ability, develop a senescence-associated secretory phenotype (SASP) producing inflammatory cytokines such as IL-6, IL-8, IL-1, IL-18, and TNF-alpha (65, 66) which contribute to the inflammaging phenomenon (60).

**Figure 1:**
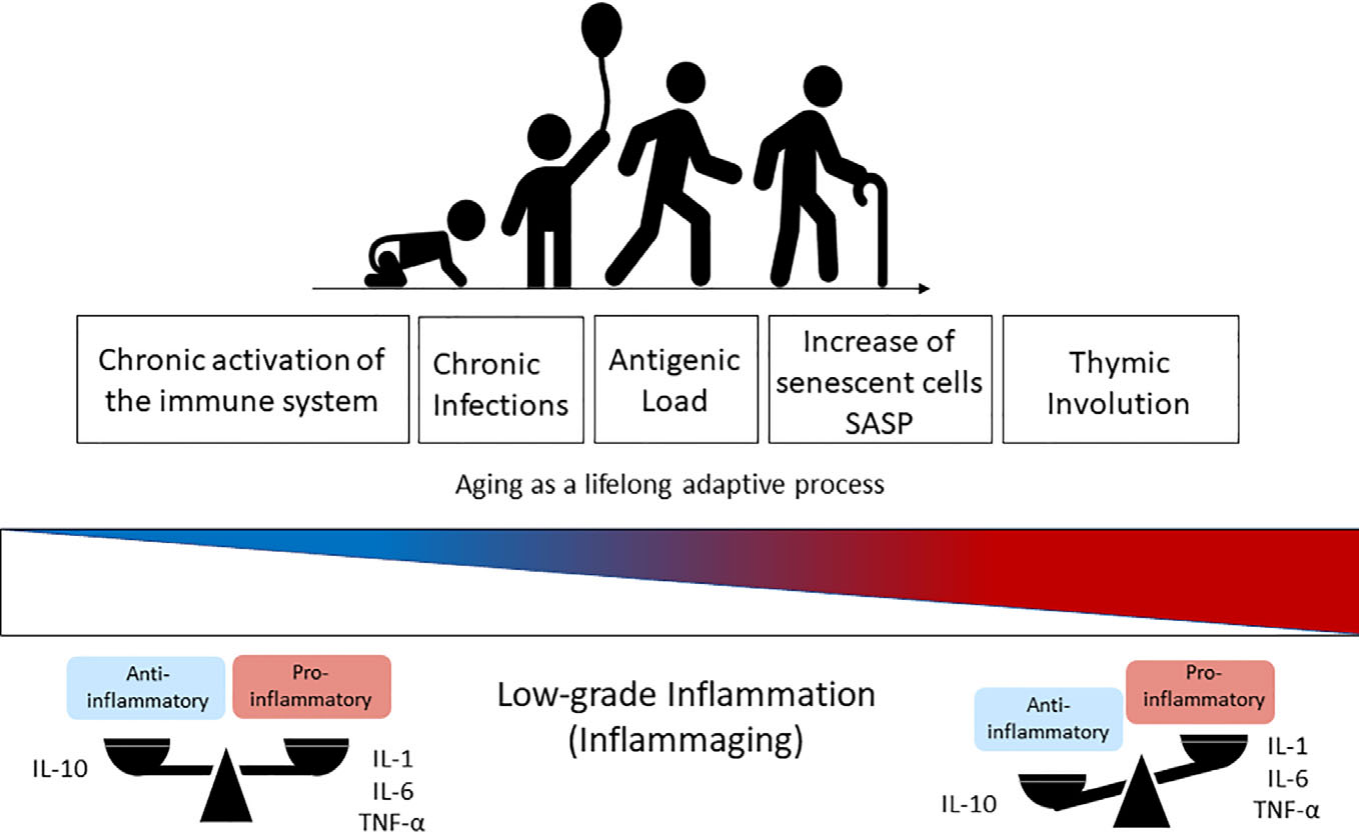
Immunosenescence is a lifelong adaptive process that occurs naturally during aging due to the exposure to antigens. The chronic activation of the immune system through time changes the health span and the balanced profile of anti-inflammatory (IL-10) *versus* pro-inflammatory mediators (IL-1, IL6, TNF-α) found in the younger ages. This balance is lost progressively with aging and results in higher levels of pro-inflammatory cytokines in elderly people, which is known as inflammaging (low-grade inflammation) and driven by senescent cells (SASP phenotype) and thymic involution. IL-1, Interleukin-1; IL-6, Interleukin-6; TNF-α, Tumoral Necrosis Factor-alfa; SASP, Senescence-Associated Secretory Phenotype.

The term inflammaging was proposed by Claudio Franceschi and coworkers (2) to name the chronic state of low-grade inflammation that is associated with aging (67). The continuous attrition caused by clinical and subclinical infections, as well as the persistent exposure to other non-infectious antigens (food, allergens, microbiota) has been correlated with chronic activation of the immune system and with the low-grade sterile inflammation that accompanies aging (41, 53). Inflammaging is characterized by the presence of high levels of pro-inflammatory cytokines such as IL-6, IL-1-beta, TNF-alpha, IL-8, IL-15, acute phase proteins (*e.g*. C-reactive protein) and can be identified in the elderly and super-elderly (centenarians) regardless of the degree of frailty (1, 5, 53). Although not fully established, some of possible causes of inflammaging include thymic atrophy, enhanced intestinal permeability, increased damage-associated molecular patterns (DAMPs), and the accumulation of senescent cells, with a consequent rise in the SASP (2, 29, 41) (**Figure 1**). In these circumstances, SASP presents as particularity the growth arrest, the resistance to apoptosis and a specific secretome (*e.g.* IL-8, TNF-alpha, IL-1-beta, IL-6, metalloproteinases, GM-CSF) that differs from inactive cells due to the preservation of metabolic activities (61, 65, 68). This phenotype can also induce DNA damage in neighboring cells by a paracrine effect and impacts on the microenvironment of the surrounding tissue impairing functioning, accelerating aging, and predisposing to age-related diseases (66, 69). Furthermore, extracellular vesicles (EVs) released from senescent cells spread pro-senescence signals that contribute to the propagation of SASP and inflammaging (70).

Interestingly, not all aged individuals have this profile, and studies on healthy centenarians and nonagenarians showed that these unique populations develop compensatory mechanisms called “immune remodeling” that allowed for the control of the deleterious effects of immunosenescence (42, 62). Data based on the stringent criteria proposed by SENIEUR protocol (9) have confirmed that senescence is not necessarily related to dysfunction of the immune system, but to a continuous immunological adaptation (42). Such process occurs heterogeneously among individuals, and it is well established that not all elderly have dysfunctional immunity, infections, chronic diseases, and frailty (24, 71, 72). Indeed, innate immune responses have been increasingly recognized for their adaptation to senescence and for their profound impact on health and longevity (24, 51). The immune remodeling is specially involved with the innate compartment. Despite the attenuation of some innate responses along the lifespan, there is a paradoxical rise in the activity of certain signaling pathways and in the production of cytokines (54). Studies have demonstrated that the cytotoxic capacity of natural killer (NK) cells is well-preserved in healthy elderly and centenarians suggesting these cells are important players in successful aging (36, 55).

Some studies also suggest that inflammaging is able to generate regulatory responses by cells with immunosuppressive phenotypes. Signals from SASP would lead to an increase in the expression and activity of the transcription factor Foxp3 in T cells and the expansion of regulatory T cells during aging (41, 56). In addition, regulatory B cells expressing IL-10 would control inflammatory effector T cells, thus aiding anti-inflammatory responses during immunosenescence (56).

Our group has studied Brazilian healthy individuals aged from 0 to 85 years and observed that the frequencies of pro-inflammatory and regulatory cytokine-producing innate and adaptive cells change during the aging process in an undulatory fashion. Although there is an increase in the frequency of cells that produce pro-inflammatory cytokines in the healthy elderly, a parallel rise in the frequency of regulatory cells could also be observed suggesting that a remodeling process takes place. IL-10-producing neutrophils and monocytes contribute to a balanced cytokine profile in these individuals (51).

In this sense, healthy immunosenescence can be seen as a result of the gradual adaptation of the organism to the deteriorative changes and the continuous stress that occurs over time (42). Indeed, according to this view, we have proposed that the body resources are continuously optimized and balanced, and successful immunosenescence consists of a potentially dynamic process of remodeling that depends on the individual's immunobiography (35). This lifelong remodeling process appears to be non-linear, as distinct fluctuations in the frequency of cytokine-producing cells throughout life was observed (51), mirroring the complex undulating changes of the blood proteome throughout life (43).

## Antigenic Load: Stressors or Immunologic Stimuli? 

Clonal mechanisms of antigen recognition can be identified only in vertebrate immune system, and they are known for being able to mount tolerance responses towards non-harmful self and quasi-self (food, microbiota) antigens and protective immune responses against dangerous antigens (toxic and infectious agents). However, the real immunological picture is not always that clear. In daily life, the immune system faces a complex mixture of misplaced and altered self-molecules resulting from damaged or dead cells, *i.e.* cell debris and organelles such as mitochondria components, that can be collectively designated as “garbage” (28, 63). The innate immune receptors for these stimuli are “degenerated”, and on many occasions do not clearly distinguish them. Thus, innate responses can trigger not only protective responses towards non-self infectious agents but can also activate inflammatory responses towards quasi-self and endogenously produced self-components (28). Balanced responses (inflammatory and regulatory mechanisms) are essential to maintain the host health under infectious circumstances; however, both types of antigenic stimuli could activate immune cells. From an evolutionary point of view, immunosenescence could be a consequence of “garbage” accumulation and of stimulation by all antigenic stressors. Given the increase in average human life span, the immune system has to tackle an extensive variety of antigens along its lifespan (3, 28, 62). This is particularly relevant for T cells, and it results in the increase of effector and memory cells (4, 44) compromising the response to novel pathogens and to vaccination (45, 52). However, considering that not all immunological stimuli are harmful to the organism, distinct antigens might lead to different impacts on health outcome (being immunosenescence the most substantial one).

The capacity of the adaptive immune system to differentiate pathogenic microorganisms from beneficial ones, preserving symbiotic microorganisms and inducing regulatory mechanisms, can be a determinant of human survival (46, 47), but again the involved molecular mechanisms are far from being clear. Immunosenescence and, consequently, inflammaging, occur as a result of chronic exposure to different antigens, but it is also known that potential stressors, such as food protein and gut microbiota, can act as a key stimuli for the development of the immune system (73, 74).

## Role of Microbiota and Dietary Antigens in the Development of the Immune System

The human body harbors trillions of microbial cells on the surface of the body (the skin and the gastrointestinal, respiratory, and urogenital tracts) (75, 76). These microbial populations, called collectively as microbiota, come to the highest density in the colon (gut microbiota) (75), and they are established since childhood (77–79). Millions of years of co-evolution created a mutualistic relationship between microbiota and human body; the microbiota improves many physiological functions of the host such as digestion and clearance of potentially pathogenic microorganisms while receiving nourishment and habitat in return (80).

The gastrointestinal (GI) tract is the largest body surface that contacts the external environment with the function of food processing. It is constantly challenged by antigens from the lumen including antigens from the diet and microbiota that will be tolerated and antigens that have to be cleared such as pathogens and toxins (76, 80). Experiments in germ-free (GF) mice showed that early life colonization by the microbiota is critical for the full development of the immune system. Germ free animals have underdeveloped intestinal mucosal immune responses, unstructured spleen and lymph nodes, smaller mesenteric lymph nodes and Peyer’s patches, an underdeveloped gut-associated lymphoid tissue (GALT), reduced frequencies of CD4+CD25+ Tregs as well as diminished levels of secretory IgA and serum IgG (81–84).

The most abundant phyla in human gastrointestinal tract are Firmicutes and Bacteroidetes, while Actinobacteria, Proteobacteria, Fusobacteria, and Verrucomicrobia are subdominant divisions (85). The diverse collection of bacteria in the human gut microbiota contributes to several physiological functions by producing short chain fatty acids and vitamins (otherwise inaccessible to humans), regulating fat storage, promoting the differentiation of various cell types, protecting the host from colonization by pathogens, and creating tonic stimuli for the development/modulation of the immune system (76). On the other hand, any intestinal dysbiosis (disturbance in microbiota composition) is associated with the onset and/or aggravation of certain diseases including some autoimmune and allergic diseases, cancer, metabolic diseases, and bacterial infections (86–90). This crucial cross-talking between the human host and microbiota can be altered through dietary habits, influencing microbiota richness and diversity and potentially impacting intestinal barrier functions and the immune system (91, 92).

Food proteins also play a critical role in this context influencing the microbiota composition and creating a daily load of antigenic components. Some of us have previously shown that mice fed a balanced amino acid-based protein-free diet (Aa-fed) from weaning up to adulthood showed local and systemic abnormalities in their immune system even though they grew normally. Aa-fed mice had underdeveloped gut-associated lymphoid tissue (GALT), low levels of secretory IgA, serum IgG and IgA, low levels of type 1 cytokines and a predominant Th2 cytokine pattern produced by cells from lymph nodes and spleen resembling a neonate profile (93). Their immune response to infectious agents such as *Leishmania major* was retarded when compared to mice fed a control protein-containing diet probably due to their immature immunological status and to the poor Th1 responses they produce (73).

In addition to their tonic properties for the immune system, it is known that exposure to these luminal antigens generates a state of specific suppression of inflammatory responses known as “oral tolerance”. Animals and humans usually tolerate the antigens that are present in their diets as well as their autochthonous gut microbiome (94, 95). Antigen presentation in such context would induce preferentially regulatory T lymphocytes (Tregs) producing IL-10 and TGF-beta with local and system modulatory effects. Tregs are major players in the tolerance response to the luminal antigens (95, 96), and oral tolerance can be seen as a process that evolved, as much as the digestive/absorptive process in the gut, to incorporate these materials as self-components.

As we age, it has been shown that changes in the composition and, remarkably, in the diversity of the microbiota are associated with health outcomes in the elderly, especially in the frailty context. Several studies have associated the gut microbiome with hallmarks of aging and immunity, including biomarkers of inflammation, immunosenescence, oxidative stress, and cardiometabolic health (89, 97–100). The gut ecosystem of centenarians differs equally from that of young adults and seventy-year old people (98). In centenarians, microbiota diversity is reduced with increased frequencies of pathobionts such as Fusobacterium, Bacillus, Staphylococcus, Corynebacterium and many members of Proteobacteriae. However, the increase of symbiotic species with reported anti-inflammatory properties, such as *Eubaterium limosum* and relatives, suggests that the composition of gut microbiota in centenarians undergoes a clear process of remodeling (98). Moreover, the gut microbiota of people characterized by extreme longevity, *i.e.* semi-supercentenarians (people who reached 105 years of age), show an increased capacity of xenobiotic degradation that likely contributes to their exceptional healthy aging (101). It is becoming clear that maintaining a health-associated microbiome is crucial to successful aging.

Interaction between dietary components and bacteria present in our microbiota also represents a source of interference with the immune system. The major products that result from bacterial fermentation of indigestible carbohydrates, also called “dietary fiber”, in the colon are short-chain fatty acids (SCFAs), including acetate, butyrate, and propionate. SCFAs are involved in the maintenance of mucosal integrity as well as colonic homeostasis: they are able to regulate leukocyte function and to influence immune responses and disease risk by signaling through GPR receptors (GPR41 and GPR43) and by inhibiting histone deacetylase (HDAC) (80, 102).

Exposure of peripheral blood mononuclear cells and neutrophils to SCFAs blocked the pro-inflammatory nuclear factor-*κ*B (NF-*κ*B) and regulated the production of cytokines (such as TNF-alpha, IL-2, IL-6, IL-17, and IL-10) eicosanoids and chemokines (*e.g.*, MCP-1 and CINC-2) (91, 103–106). In this context, inhibition of HDACs by SCFA fosters an anti-inflammatory tolerogenic milieu indicating that the microbiota acts as an epigenetic regulator of body homeostasis (103).

Some dietary components, such as B vitamins and vitamins A, D, K, and E can be synthesized by the gut microbiota and have a key role in reducing inflammation, regulating energy metabolism, enzymatic functions important for gene expression and immune response regulation throughout the life course (91, 107, 108). Interestingly, both zinc deficiency and dysfunction of the immune system are accompanied by impaired immune responses and systemic low-grade chronic inflammation in aged individuals (109–112). Sodium chloride (NaCl) is a salt and a micronutrient that also mediates immunological effects. In high concentrations, NaCl can induce alterations of gut microbiota composition, modifications of gut permeability, and inflammation in the gut mucosa, increasing the susceptibility to colitis development (113, 114). As an inflammatory stimulus, consumption of high-salt diets may interfere with the aging process.

Microbiota and dietary components are clearly innocuous natural antigens that promote immunological development at early age and regulatory immune responses throughout life. However, pathogens cannot be considered as tonic agents. Contacts with clinical and sub-clinical infections usually lead to inflammatory responses, and they may be considered as important stressors that continuously impinge on our immune system (48, 115).

## Hygiene Hypothesis—Infections as Beneficial Immunological Stimulation in Childhood

The “Hygiene Hypothesis” and the “Old Friends Theory” advocate that childhood infections and microbiota provide immunoregulatory mechanisms that shape our immune system to cope with the continuous exposure of the body to antigens. The lack of contact with these infectious agents, due to high hygienic conditions (we became too “clean”), increases the incidence of atopic diseases, autoimmune and some chronic inflammatory disorders (116–119).

In the past few years, a large scientific effort has been focused on understanding how microbiome and parasites modulate the human immune system, especially how exposure to these antigens impacts on the incidence of inflammatory and age-related diseases (119–121). The scope of disorders affected by contact with different microbiomes and food components has been enlarged lately including allergies, autoimmunity, inflammatory bowel disease, celiac disease, food allergy, vascular disease, cancers, and inflammation-associated psychiatric disorders (117, 119, 122). However, there are pathogens such as respiratory syncytial virus (RSV) or rhinovirus that are not protective in any scenario and are usually associated with a high susceptibility to develop wheeze and asthma in children and adults alike (123, 124). In addition, the human microbiome is itself subjected to the influence of several related variables including microbial exposure, diet, lifestyle, medication, parasite infection, among others, and this network of influences may also be reflected in the immune system operation at steady state and in the onset of immune-mediated diseases such as allergy (125–129).

Therefore, the age when the contact with these antigens occurs is a determinant factor for the later immunological consequences they trigger. The antigenic load represented by food and microbiota as well as some parasitic antigens at early time in life has long lasting beneficial effects in the immune system contributing to the robustness of the regulatory immune mechanisms that operate in adults and old individuals (49, 130). Young adults still have a large repertoire of lymphocytes with high diversity and plasticity being able to mount proper immune responses when challenged by a variety of new antigens. In spite of that, it is not clear how durable are the effects of antigenic stimulation at this time point in life. On the other hand, the immune system of the elderly is less capable of properly dealing with new antigens since aging is associated with an increasing loss in repertoire diversity. At this late period of life, introduction of antigenic novelties, even if they are microbiota or dietary components, might represent a threat rather than a tonic regulatory stimulation (49).

The pleiotropy hypothesis of aging proposed by George Williams in 1957 presents an evolutionary perspective to interpret these discrepancies in immunological behavior when facing antigenic stimulation throughout life. It suggests that genes that evolutionarily resulted beneficial at young age became detrimental at old age, a period that was largely unpredicted by evolution. Such genes would be favored by natural selection by enhancing fitness early in life, a period when selection is stronger, even if they cause the aging phenotype to emerge (131). Today, it is generally accepted that antagonistic pleiotropy is common if not ubiquitous, implying that also a number of other molecular and cellular mechanisms of aging such as immunosenescence and inflammaging can be interpreted within such a conceptual framework.

## Genetic and Epigenetic Factors as Major Determinants of Immunosenescence 

Aging is a natural phenomenon that affects individuals differently. While centenarians are clear examples of resilience against the detrimental effects of antigenic attrition during aging (132), some individuals present signs of aging and age-related diseases early in life. Studies on centenarians who present high levels of inflammatory mediators suggest that inflammaging is compatible with longevity (17, 132). These different performances of individuals facing aging and inflammaging may have strong genetic and epigenetic determinants (133, 134). The human lifespan is in part heritable; another part of the aging process is related to environmental factors such as injuries, lifestyle, socio-economic and education levels, and work activities (133). Heritability can increase from nonagenarians to centenarians (100+), semi-supercentenarians (105+), and supercentenarians (110+), and people who reach above 90 years of age appears to have stronger genetic basis for their longevity (135, 136).

Epigenetic factors mediate the relationship between the environment and the genome, and they are also involved in aging and age-related diseases (137). One of the most important epigenetic factors, DNA methylation, is known to influence the outcome of aging. DNA methylation is strongly related to unique individual environments, and only a small fraction of DNA methylated sites associates with familial factors (genetic or shared environment) (138). In elderly twins, for instance, a different profile of methylated CpGs is observed over time (133). Many of the methylated genes are involved in the regulation of the immune system, especially of lymphocytes (139). Interestingly, the geographical location can alter DNA methylation patterns in family individuals supporting the hypothesis that inflammaging and immune modulation in aged individuals may vary among regions and countries.

Lifestyle and environmental factors are strongly involved in the basis of longevity and aging. Regarding dietary influences, animal studies on caloric restriction (CR) showed that mitochondrial-derived free radicals generated during ATP production are inducers of cellular senescence and aging. Indeed, caloric restriction in rodents is able to increase their life span by up to 50% indicating a correlation between oxidative stress in the mitochondria and life span (140). A randomized clinical study conducting CR during two years in people demonstrated that CR effectively controlled energy expended and oxidative stress, improving life expectancy (141). Caloric restriction and rapamycin treatment are also involved in anti-aging process, increase in lifespan, improvement of physiological functions, and reduction of pathology (137). On the other hand, a chronic inflammatory condition such as obesity is associated with decreased size of telomere length and also with increased oxidative stress in cells, leading to early aging and dementia (142, 143). Regular physical activity can be associated with decreased levels of oxidative stress and pathological inflammation. A recent study in twins revealed that sports are related with differences in telomere lengths between them (144). Another factor that impacts life expectancy is work behavior. People who work in night shifts or have irregular hours of work for more than 10 years had accelerated epigenetic age with differentially methylated CpG sites across their epigenome, including in genes for circadian rhythm (145).

There are genetic variations in certain proteins that became markers of longevity. One of them affects the uncoupling proteins (UCP1, UCP2 and UCP3) that belong to the family of mitochondrial transmembrane carriers and are regulators of the respiratory process in mitochondria. These proteins are able to decrease ATP-generation and ROS production by dissipating the proton gradient of the inner mitochondrial membrane resulting in the increased longevity observed during CR (146). Variations on sirtuin genes (*SIRT1*, *2*, and *3*) were also reported to influence the mitochondrial functionality and longevity (147, 148). *SIRT1* gene is involved in decreased oxidative stress and inflammatory response, and *SIRT3* is a mitochondrial deacetylase that reduces ROS production. Both genes are downregulated in the elderly and the activation of micro-RNA-9 (miRNA-9) can improve their functions resulting in decrease aging (149). Another study showed the involvement of micro-RNAs (miRNAs) in the development of the age-related Alzheimer’s disease. Two miRNAs, mi-146b-5p and miR-15b-5p, were identified in a cohort as related to innate immune responses and regulation of cell cycle (150). A recent genome-wide analysis of miRNAs in centenarians and nonagenarians showed different clustering between the long-aged individuals and the younger controls. Cancer related proteins such as p53 and others were shown to be potential targets of these miRNAs indicating that tumor suppression and maintenance of genomic integrity are critical events during aging (151).

Genes related to cell cycle regulation and telomere length, such as *P21, FOXO3A, TERT, and TERC*, have also been described as associated with longevity (133). The cell cycle inhibitor *P21* or *CDKN1A* gene is induced by stress responses and inflammation during senescence, and it is implicated in the upregulation of several age-related genes (35, 152, 153). The *FOXO3* gene can have more than 100 SNPs, and some of them are associated with very long life span (154).

In fact, some individuals have more susceptibility to age-related diseases as dementia and cardiovascular diseases (134). Alzheimer’s disease, diabetes mellitus, and cancer are common diseases among Western old people probably due to the presence of genes that predispose to those health conditions. On the other hand, some individuals from the same region are resistant to those aged related diseases probably because they lack other genes involved in disease development. The *APOE* gene, for instance, has different variants that are associated with high susceptibility for cardiovascular and Alzheimer’s diseases (155–157). Another set of polymorphisms associated with age related diseases is at *FTO* (fat mass and obesity associated) gene, which are involved in increased morbidity and mortality due to increased adiposity and obesity in humans (156, 158). Polymorphisms (SNPs) in *FTO* gene have been recently described as associated with increased risk for Alzheimer’s disease (159). *FTO* is also involved in cell cycle, and its silence prolongs G1 phase, reducing cell proliferation (160). SNPs in *SDC4* gene were investigated for their association with higher longevity in a cohort above 64 years old. *SCD4* encodes a transmembrane protein, Syndecan 4, that is associated with microglia activation during neuroinflammation. *SDC4* SNPs might have influence on lipid metabolism during aging, and *SDC4* gene SNP rs1981429 was negatively associated with longevity in the group between 64 and 85 years old. The same association is also observed for high triglyceride level and for low levels of LDL cholesterol. On the other hand, SNP rs2251252 seems to be associated with longevity and with high levels of LDL (161).

A simple conclusion from the genetic studies demonstrating a variety of polymorphisms involved in immunosenescence underscores the great importance of studying aging across distinct genetic backgrounds and distinct ethnical groups. A study conducted during 18 years in UK showed that the healthy condition of the offspring is associated with parental lifespan. In addition, the lower incidence of cardiovascular diseases, cancer, and reduced cognitive decline in certain populations is associated with higher parental and offspring survival (162). In a Spanish cohort, researchers observed that homozygosis in 192bp allele of *IGF-1* gene is a marker of healthy aging. Polymorphisms in this gene could be related to obesity and several derived conditions such as metabolic syndrome, cardiovascular diseases risk, cachexia and premature death (163). A study in centenarians from Italy showed five genes (*HRAS1, SIRT3, TH, INS*, and *IGF2*) associated with longevity. However the same result was not observed in individuals from Germany (148). Therefore, different genes may be associated with longer life in distinct populations. Although centenarians usually display the same longevity of their families (164), they would show distinct ways to attain a longer and healthy life depending on their geographical location, genes and lifestyle.

## The Role of Inflammaging in Endemic Areas for Chronic Infectious Diseases

NK cytotoxicity has been described as a biomarker of immunological remodeling, healthy aging as well as longevity, and it seems to compensate for the changes/deficiencies occurring in other immune functions lost by lymphocytes during immunosenescence (42, 55, 62). Cumulative evidence in the last two decades identified a well-preserved NK cell activity in both healthy elderly individuals and centenarians (57–59, 165). Kaszubowsaka and coworkers demonstrated that the expression of TNF-alpha by non-stimulated cells was significantly higher in both CD56^dim^ and CD56^bright^ NK cells of aged individuals when compared to young ones. Moreover, CD56^dim^ NK cells of the oldest were responsive to the IL-2 stimulation (59). As previously described, the increase of the inflammatory microenvironment associated with aging (6, 55, 166–168) may lead to degenerative and inflammatory chronic diseases (14, 20, 44, 50, 63). However, this immune profile can be important for an efficient response against infectious parasitic diseases, especially for individuals living in endemic areas.

In this context, in countries where the elderly population live in endemic areas for infectious diseases such as Chagas disease, leishmaniasis, and schistosomiasis, the presence of an inflammatory reactivity can favor these individuals against the constant challenges. Few studies on the effects of aging and specifically of inflammaging in Brazilian populations are available. Our groups are part of the few in the country working on the topic, and we have already examined the cytokine/chemokine profile of elderly from Belo Horizonte (51), Governador Valadares (89), and Bambuí (169, 170) in Minas Gerais State. Although we observed changes in the profile of pro-inflammatory *versus* regulatory cytokines throughout life in all these locations, they did not show a simple increase in inflamming-related cytokines.

It is true that life expectancy has increased in industrialized as well as in developing countries. Nevertheless, the medical challenges to deal with the aging population in these regions are very distinct. Considering that chronic infectious diseases are still prevalent in most developing countries (171), understanding the clinical outcomes of tropical diseases in elderly patients and how frailty is related to them will help to define healthy aging in different scenarios.

## Chagas Disease

Although several studies have shown the importance of immune response in Chagas disease progression, the mechanisms underlying the severe forms of this disease are still elusive. The balance between inflammatory and modulatory cytokines towards an anti-inflammatory profile contributes to the control of the disease and to the development of its milder forms. Conversely, severe diseased patients with the cardiac form developed a Th1-specific immune response with inflammatory infiltrate and tissue damage (172–174). Our group studied elderly adults from an endemic area for Chagas disease analyzing the correlation between serum levels of cytokines and chemokines, *Trypanosoma cruzi* infection, and cardiac abnormality (175). When compared to healthy controls, Chagas disease patients had higher circulating levels of IL-1-beta, CXCL9, and CXCL10 and lower levels of CCL5 than healthy subjects. Interestingly and in contrast with control individuals, levels of CXCL9 and CXCL10 continuously increased with age indicating that these two chemokines are strong markers of immunosenescence in the elderly with Chagas disease (175).

## Leishmaniasis

Visceral leishmaniasis (VL) is a neglected re-emerging chronic infectious disease in tropical and subtropical regions (176) where it is related to poor access to health care and poverty. Countries like Brazil have reported a high incidence of new cases annually (177) although it is not clear whether all cases result from recent infection or from reactivation of latent infection in patients that have chronic immunosuppressive conditions such as HIV (178) and organ transplantation (179). The rate of positive Leishmania skin test results in some areas of Brazil is extremely high in the elderly, and this might become a relevant geriatric issue (171). In Teresina, for instance, the capital of a state in the northeast Brazil, 50% of tested individuals were positive, and prevalence increased with age (180) suggesting that some of these individuals were experiencing a reactivation of a previous infection. Moreover, the overlapping of two chronic infections such as VL and HIV is reported to create an environment of persistent cellular activation inducing senescent/exhausted lymphocytes, affecting the generation of new T cells and accelerating immunosenescence (181, 182). Indeed, the thymus of patients living with HIV presents alterations in the lymphoid and stromal compartments as well as in the generation of the V-beta repertoire of T lymphocytes (183) that are typical of aging. In addition, HIV infection potentially contributes for the inflammatory immunopathogenesis of VL and, at the same time, impairs the effector immune responses to antigens, including Leishmania (184).

## Schistosomiasis

More than 230 million people are infected with schistosomiasis worldwide (185) causing a huge impact in the quality of life of affected individuals (186). Our group has studied individuals from schistosomiasis endemic areas in Minas Gerais State and showed a clear correlation between well preserved mechanisms of innate immunity and the absence of infection in elderly subjects. We observed an increase in the frequency of IFN-gamma^+^CD16^+^NK cells in non-infected elderly individuals when compared to *Schistosoma mansoni* infected ones (187). Moreover, it was observed that non-infected elderly individuals present an increase in the frequency of the natural killer (NK) cells, macrophages, and dendritic cells expressing Toll-like receptors (TLR)-1, suggesting that, in endemic areas, remodeling of innate immunity mechanisms may have a protective role that could compensate for the aging-related decline in T-cell responses (188). In addition, the augmented frequency of T cells with a regulatory phenotype (Foxp3^+^CD25^+^CD4^+^, LAP^+^CD4^+^, and IL-10^+^CD4^+^) observed in infected aged individuals from these endemic areas (when compared to non-infected ones) may have two consequences: they may hinder the development of protective immune responses but they also explain the absence of severe hepatosplenic clinical form of the disease during chronic infection in these individuals (189). Together, these results support the hypothesis that an inflammatory innate immune response in parallel with the decrease of regulatory mechanisms (as observed in non-infected individuals) can induce a protective immunity in elderly individuals from schistosome endemic areas. Although this can be seen as a desirable “protective profile”, it also suggests that remodeling in these regions of high antigenic load (infectious agents) occurs at the expense of immune regulatory mechanisms which are important for controlling inflammaging.

## Accelerated Aging

**Figure 2:**
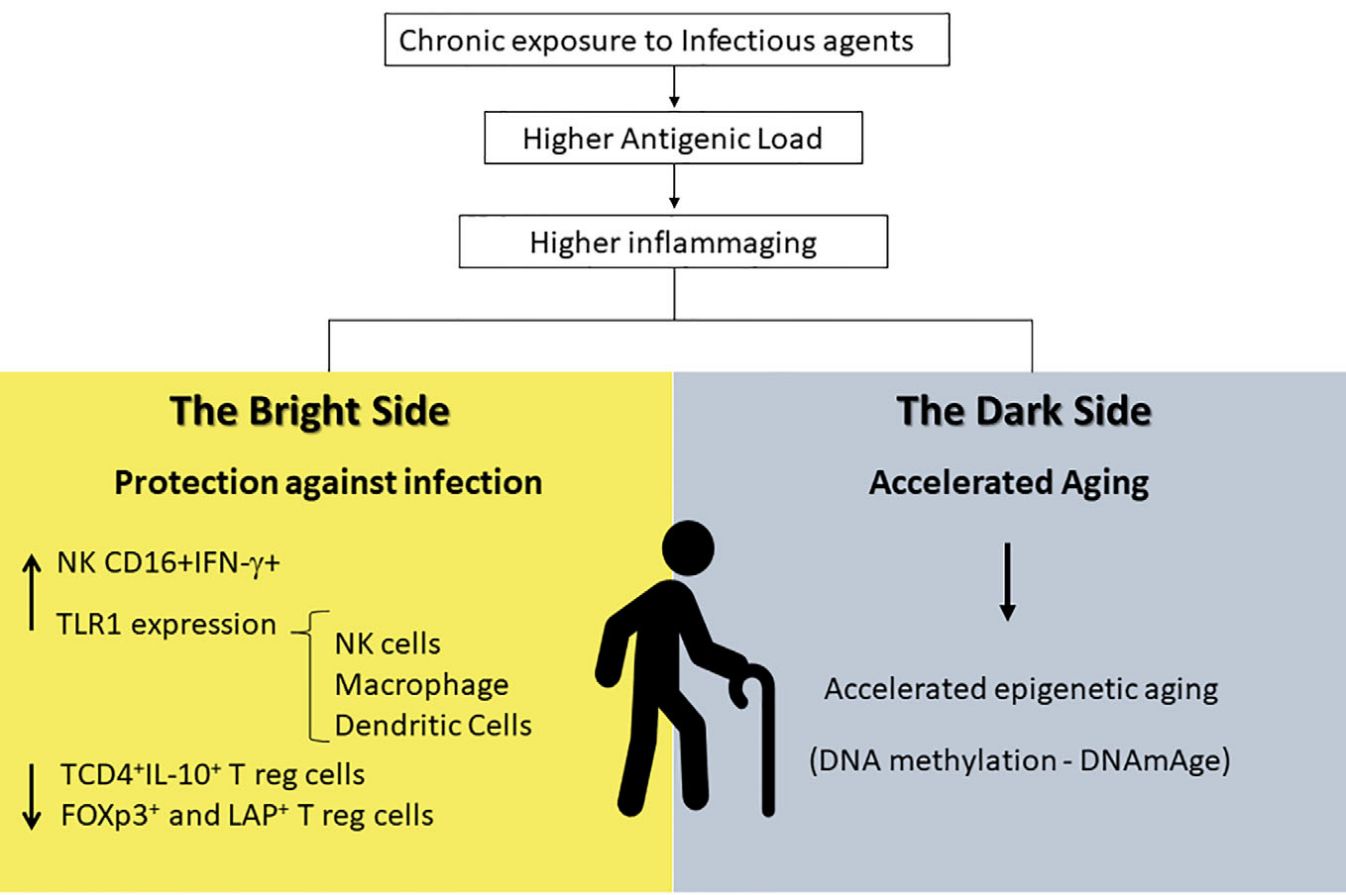
Inflammaging is a distinct and exacerbated process in endemic areas that provides higher antigenic load and exposure to chronic infections. It seems to have a bright side resulting in protection against infectious antigens that can be explained by the increase in INF-*γ*-producing NK cells and in TLR-1 expression in innate immune cells (macrophages, NK, and dendritic cells) and by the decrease in regulatory T cells in general. At the same time, it has a dark side that might be responsible in accelerating aging due to epigenetic mechanisms. NK, Natural Killer; INF-*γ*, Interferon-gamma; TLR-1, Toll-Like Receptors-1.

It is reasonable to believe that inflammaging could play a role in protective immunity in endemic areas for infectious disease. If one takes the premises of the hygiene hypothesis, exposure to infectious agents since childhood may induce both protective effector immune responses as well as robust life-long regulatory mechanisms that would prevent the spill-over effects of inflammaging causing degenerative diseases and frailty. However, inflammatory responses could have other consequences such as acceleration of the aging process itself. This may come as a price to pay for protective immunity. A hallmark of immunosenescence is the reduction in the output of naïve T and B cells and the increased frequency of memory and effector lymphocytes as a consequence of thymic involution. The continuous exposure to natural antigens (microbiota, food proteins, allergens) and the antigenic stress caused by clinical and sub-clinical infections may lead to inflammaging, degenerative diseases and frailty in senescence (190, 191). A wide range of age-related diseases including diabetes, auto-immune diseases, osteoporosis, sarcopenia, neurodegeneration, and atherosclerosis has a common inflammatory pathogenesis (53, 190). Therefore, it is expected that individuals exposed to a higher burden of antigen load would present accelerated immunosenescence, higher morbidity and mortality. If we also take into account the reduction in immunoregulatory mechanisms required in endemic areas to preserve protective immunity as reported earlier (189), and the fact that some components known to counteract cellular stress such as heat shock proteins are also diminished in the elderly (192), the accumulation of stress attrition during aging would be particularly deleterious. Although inflammatory responses mounted by individuals living in these areas are directed towards protective immunity, being infected or non-infected, the chronic exposure to infectious stressors may accelerate aging and predispose them to frailty (**Figure 2**).

After chronic and prolonged replication, cell senescence occurs as a natural part of the aging process but can potentially be accelerated in response to a variety of insults. Stress-induced premature senescence is a result of cytotoxic stimuli such as oxidative stress, proteasome inhibition or activation of *RAS*, and *Myc* oncogenes by tumorogenic agents (61, 69). These various stressors can induce cell cycle arrest, DNA damage, heterochromatin formation, increased senescence-associated beta galactosidase (SA-beta Gal) activity, expression of the cell cycle inhibitors, and the secretion of pro-inflammatory cytokines and proteases as part of the senescence-associated secretory phenotype (SASP) (65, 68). Senescent immune and non-immune cells are critical for the inflammaging phenomenon.

Some pathological conditions act as chronic stressors inducing premature senescence. Chronic Obstructive Pulmonary Disease (COPD) is considered a condition of accelerated lung aging, and senescent cells with shortened telomers have been identified in emphysematous lungs (193). Some reports also exist on the role of cancer and cancer treatment as stressors that could accelerate aging (194). On the other side of the spectrum, the most common natural process that accelerates epigenetic aging of blood cells is menopause (195).

Down syndrome is also associated with premature aging. Using DNA methylation (DNAmAge) as a measurement for biological age, Bacalini and coworkers identified an epigenetic signature of DS that sustains a link between developmental defects and disease phenotype, including premature aging (196). They found that methylated regions (DMRs) displayed a genome-wide distribution although they were enriched on chromosome 21 in genes involved in developmental functions, including neuronal (*NCAM1*), embryonic (*HOXA* family), and hematological (*RUNX1* and *EBF4*) development as well as regulation of chromatin structure (*PRMD8, KDM2B, TET1*). Interestingly, Biagi and coworkers reported alterations in gut microbiota of individuals with Down syndrome towards an overall immunomodulatory profile, when compared to that of healthy controls (197). This suggests that gut microbiome may counteract the genetic determined acceleration of immunosenescence in Down syndrome individuals.

Since infections are stressors and stimulators of immune cells, chronic infections can be potential stressors able to induce aging acceleration. In HIV infection this possibility has been already investigated. Growing evidence reveals a premature aging phenotype that accompanies HIV-infected patients (186, 187, 198). Thymic alterations in the development of T cell repertoire as well as increased frequency of senescent/exhausted T cells are part of the phenotype (182, 183). These patients are exposed to several stressors including the virus itself, antiretroviral drugs, and very often, drug abuse. The fact that they suffer from a number of comorbidities commonly associated with frailty in the elderly such as diabetes, renal failure, atherosclerosis, neurological deficits, and osteoporosis also confirms the age acceleration hypothesis (198).

Other infectious diseases caused by virus might have an additional effect on aging and immunosenescence, especially in poor countries. In this context, human cytomegalovirus (CMV), a herpesvirus highly frequent worldwide, has a well-known impact in immunosenescence. Its prevalence is strongly associated with lower socioeconomic conditions and ethnicity, as verified by the National Health and Nutrition Examination Survey (NHANES) III conducted in the United States. A significant racial and socioeconomic disparity was found in CMV seroprevalence in children and young adults (199). The prevalence of CMV in German adults is 56.7% (200); it approaches 80% by the age of 70 years in Northern Europe (201); and it ranges from 8.7 to 99.2% in the MENA region (Middle East and North Africa) according to a systematic review (202).

The relevance of CMV in immunosenescence has been an important topic of discussion since the publication of OCTO and NONA studies, when it was defined as a part of the immune risk profile (IRP) and associated with increased mortality in older ages (14, 203, 204). Nevertheless, the BELFRAIL study conducted with very elderly individuals did not find a relationship between CMV infection and mortality in Belgium (205). When it comes to HIV patients, the presence of CMV co-infection seems to boost immunosenescence, not only by increasing late-differentiated CD8+ T cells regardless of chronological age, but also by promoting an accelerated telomere erosion in that same subset of cells (206).

The most pronounced effect of CMV in the immune system is memory inflation, a term used to describe the expansion of memory T cells with the accumulation of late-differentiated CD8+ effector cells (TEMRA) that re-express CD45RA, are considered senescent cells and increase with age (30, 207). Other possible contributions of the virus to senescence are the increase in inflammatory mediators and the elevated risk for age-associated morbidities, such as cardiovascular diseases, cancer, atherosclerosis, diabetes, and Alzheimer’s disease (204, 206). Therefore, CMV infection seems to be a driving force accelerating immunosenescence due to its impact in T cell senescence and inflammaging. Epidemiological studies conducted in the US and in England showed a clear association between positive serology for CMV and cardiovascular disorders such as hypertension and ischemic heart disease (208–210). Cytomegalovirus can infect endothelial cells where they replicated and recruit neutrophils, monocytes, CD4, and CD8 cytotoxic T cells causing vascular damage (211, 212). The augment in the frequency of CD4+CD28− T cells, which are highly cytotoxic and producers of pro-inflammatory cytokines such as INF-gamma and TNF-alpha, seems to play a key role in the development of autoimmune and cardiovascular diseases (213). According to recent reports, increased levels of CD4+CD28− T cells are highly associated with CMV seropositivity, while aging lightly contributes to this change (214, 215). Furthermore, since CMV infection is linked to a range of cardiovascular and metabolic disorders, it might also influence negatively the clinical outcome of SARS-Cov-2 infection although positive serology for CMV alone has not been confirmed as an independent risk factor (216).

CMV-seropositive elderly individuals have a higher chance of developing age-associated chronic inflammatory diseases, but it remains unclear whether this latent infection has only a negative impact in the longevity (206). A study by Bajwa and coworkers (217) addressed the polyfunctionality of T cells (*i.e.*, several T-cell effector functions), a relevant quality for protection against virus and to vaccination, evaluating CMV-specific CD4+ and CD8+ T-cell responses to 19 different CMV target proteins in young and old volunteers. They showed that CMV specific T-cell polyfunctionality was not decreased in the healthy elderly, but it was reduced in the oldest-old group raising the question whether polyfunctional T cells in older people were necessarily associated with protection and longevity. Along the same line, Terrazini and coworkers (218) showed that most of CMV-specific CD4+ T cells have anti-inflammatory properties and may mediate a beneficial effect in aged individuals regarding cardiovascular disorders. The authors showed that CMV-specific induced regulatory CD4+ T cells (iTregs) are at high levels in older individuals, and they correlate with levels of CD8+ effector cells. A significant association between these CMV-specific T-cell subsets (CD4+ and CD8+), arterial blood pressure and vascular stiffness was found. Most of the iTreg cells expressed Foxp3; they suppressed antigen specific as well as non-specific proliferation and attenuated the inflammatory response as well as the cardiovascular pathology caused by CD8+ T cells.

Many studies have suggested that CMV effects in the immune system are age dependent. At early age, the chronic CMV infection might serve as a trigger to maintain the immune system in constant alert, enabling rapid recall responses and enhancing heterologous immune responsiveness, particularly prior to reproductive age (207, 219, 220). The inflammatory process triggered by this chronic infection can stimulate the maturation of the immune system and improve responses to homologous antigens. Additionally, there is evidence suggesting that infected young individuals present a better response to influenza vaccination (221). However, individuals that become seropositive at older ages were reported to have impaired response to vaccination (221–224). The cytokine storm triggered by CMV infection seems to compromise immune responses to influenza vaccine for instance (201). Therefore, from an evolutionary perspective, the detrimental effects of CMV infection during immunosenescence can be seen as a later consequence of its tonic role in immune responses earlier in life (220).

In endemic areas for chronic infections such as Chagas disease, leishmaniasis, schistosomiasis, and leprosy, individuals are usually exposed to infectious agents during their lifetime. In many regions in Brazil, such as the northeast of Minas Gerais, these diseases are a result of poor sanitary and economic conditions, and they co-exist increasing the burden for the immune system of the individuals who live there (189). Preliminary results from Ana Faria’s group (D. Durso and coworkers, unpublished data) show that individuals from a city located in one of these areas, Governador Valadares in Minas Gerais, present an accelerated epigenetic aging phenotype as measured by DNA methylation (DNAmAge) as described by Horvath and coworkers (225).

Furthermore, we cannot rule out the possibility that the immunosenescence phenotype would be a risk factor for severe outcomes in viral infections such as COVID-19. An important characteristic of the SARS-Cov-2 infection is the pattern of high-risk groups reaching mainly individuals with underlying comorbidities such as diabetes and cardiovascular diseases and elderly people. In China, case-fatality rate was 0.4% in 40–49-year-old patients, 1.3% in 50–59, 3.6% in 60–69, 8.0% in 70–79 and reach 14.8% in >80-year-old patients (226). Similar findings were reported in Italy where case fatality rates were 12 and 20% among those aged 70–79 years and 80 years and older, respectively (227). Senescence-associated decline in immune function observed in aged people (inflammaging resulting from increased innate cytokine secretion, decline in effector and regulatory CD4+ T function and increased frequency of exhausted/senescent CD8+ T cells) may have a critical role in the development of lung and microcirculation damage and severe respiratory syndrome in SARS-Cov-2 infected elderly. This possibility has been speculated by few authors. Alterations such as lymphopenia have been identified as a tread linking COVID-19 and frail elderly. Indeed, these groups of individuals share a decline in the numbers of CD4/CD8 T cells but not of B cells (228). Others propose using biomarkers of biological age as predictors of disease severity by SARS-Cov-2 (229), and also that reversing immunosenescence would impact in the outcome of COVID-19 (230). Finally, it has been suggested that elderly with pre-existing but clinically silent CMV infection might be particularly susceptible to the severe COVID-19 since infection with cytomegalovirus is known to trigger the cytokine storm, reduction in naïve T-cell accumulation of terminally differentiated CD8+ T cells and impaired immune responses to vaccination (201). These propositions are worth further investigation.

## Conclusions

The rapid aging of the population in developing countries is an unprecedent demographic phenomenon that is accompanied by the high prevalence of chronic infectious diseases among individuals who live there. This process represents a public health problem and a biological challenge. As part of the aging process, immunosenescence triggers several alterations in the immune system resulting in poor response to infection and increase in inflammation. However, inflammaging can be associated with remodeling mechanisms as the ones observed in healthy elderly. Although the concept of healthy aging has been proposed initially to describe the European individuals who reach advanced age free of the inflammatory consequences of immunosenescence, it is now clear that this concept must be broadened to encompass distinctions related to the role of inflammaging and remodeling according to genetic, epigenetic, environmental, and cultural scenarios in which the aging process takes place. Understanding these geographical differences in immunosenescence could provide a better understanding of age-related changes as well as their treatable effects. It will aid in the prevention, diagnosis, and treatment of some age-related dysfunctions as well as infectious diseases in the elderly.

## Author Contributions

MAB helped in writing and organizing the first draft of the manuscript. FC-F, GS-N, GC, ES, ST, AT-C, OM-F, TM, NN, PB, and RS helped writing specific sections of the manuscript. CF helped with the original concepts and ideas as well as revising the writing of the manuscript and AMCF planned the original concepts, helped writing, and was in charge of the final version of the manuscript. All authors contributed to the article and approved the submitted version.

## Funding

This work had financial support from Fundação de Amparo à Pesquisa do Estado de Minas Gerais (FAPEMIG, Brazil) and Pró-reitoria de Pesquisa of UFMG (PRPq-UFMG). Some of the authors are recipients of fellowships (AMCF, OM-F, AT-C, TM) from Conselho Nacional de Desenvolvimento Científico e Tecnológico (CNPq, Brazil).

## Conflict of Interest

The authors declare that the research was conducted in the absence of any commercial or financial relationships that could be construed as a potential conflict of interest.
